# 3D Printing of Piezoelectric Barium Titanate-Hydroxyapatite Scaffolds with Interconnected Porosity for Bone Tissue Engineering

**DOI:** 10.3390/ma13071773

**Published:** 2020-04-09

**Authors:** Christian Polley, Thomas Distler, Rainer Detsch, Henrik Lund, Armin Springer, Aldo R. Boccaccini, Hermann Seitz

**Affiliations:** 1 Chair of Microfluidics, University of Rostock, 18059 Rostock, Germany; hermann.seitz@uni-rostock.de; 2 Institute of Biomaterials, Friedrich Alexander University Erlangen-Nuremberg, 91058 Erlangen, Germany; thomas.distler@fau.de (T.D.); Rainer.Detsch@fau.de (R.D.); aldo.boccaccini@fau.de (A.R.B.); 3 Leibniz Institute for Catalysis at the University of Rostock, 18059 Rostock, Germany; henrik.lund@catalysis.de; 4 Electron Microscopy Centrum, University Hospital Rostock, 18057 Rostock, Germany; armin.springer@med.uni-rostock.de; 5 Department Life, Light & Matter, University of Rostock, 18059 Rostock, Germany

**Keywords:** biomaterial, piezoelectric, bone, 3D printing, barium titanate, bioceramic

## Abstract

The prevalence of large bone defects is still a major problem in surgical clinics. It is, thus, not a surprise that bone-related research, especially in the field of bone tissue engineering, is a major issue in medical research. Researchers worldwide are searching for the missing link in engineering bone graft materials that mimic bones, and foster osteogenesis and bone remodeling. One approach is the combination of additive manufacturing technology with smart and additionally electrically active biomaterials. In this study, we performed a three-dimensional (3D) printing process to fabricate piezoelectric, porous barium titanate (BaTiO_3_) and hydroxyapatite (HA) composite scaffolds. The printed scaffolds indicate good cytocompatibility and cell attachment as well as bone mimicking piezoelectric properties with a piezoelectric constant of 3 pC/N. This work represents a promising first approach to creating an implant material with improved bone regenerating potential, in combination with an interconnected porous network and a microporosity, known to enhance bone growth and vascularization.

## 1. Introduction

Biological electricity appears to play a vital role in bone homeostasis, and particularly in bone remodelling and repair [1]. In the early 1960s, Japanese scientists found bone tissue to be a piezoelectric material, due to the displacement of hydrogen bonds in the polypeptide chains of collagen [2,3]. Applied stress, as developed from the motion of the body itself, results in a change of polarization in the material and induces an electrical dipole. Recent studies underline the results of Fukada and Yasuda (1957) and expanded their research through the detection of piezoelectricity in hydroxyapatite [4,5]. Therefore, many research groups have considered how to utilize the intrinsic bioelectric properties with a new category of electrically active implants [6,7,8,9]. The implants could be used as sensors, actuators for energy harvesting or as new biomaterials that mimic the electrical behavior of bones. Such implants could be promising candidates for advanced bone repair strategies. One of the main concerns of biomedical implants is the safety and biocompatibility of the utilized biomaterial. While, lead zirconate titanate (PZT) ceramics show excellent piezoelectric properties with a high piezoelectric constant from 200–350 pC/N, they cannot be used as a biomaterial in tissue engineering applications, due to their cytotoxicity [10]. Promising candidates are lead-free piezoceramics, such as potassium sodium niobate (KNN), lithium-doped potassium sodium niobate (LKNN) and barium titanate (BaTiO_3_) that are being investigated, while BaTiO_3_ represents the most studied lead-free piezoceramic regarding the use as a biomaterial [8,9,11,12,13]. The advantages of using BaTiO_3_ as a scaffold material for bone tissue engineering have been reported through in vitro studies, as well as in vivo studies on small animals [8,9,13].

**Figure 1:**
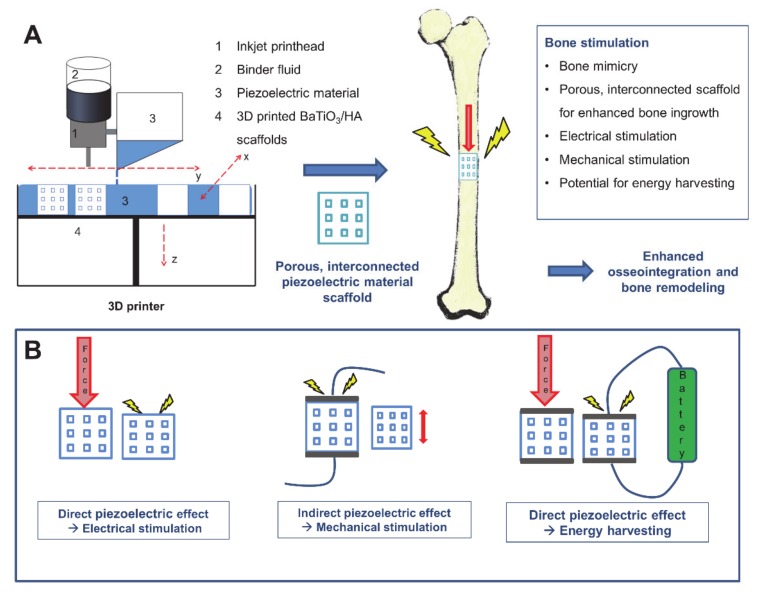
Overview of 3D printing of piezoelectric materials for bone stimulating implants. (**A**) shows exemplarily the binder jetting process used for the fabrication of piezoelectric scaffolds. (**B**) indicates different applications of the piezoelectric effect for bone stimulation. Piezoelectric implants have the potential to stimulate electrically (direct piezoelectric effect), mechanically (indirect piezoelectric effect) or could be used as an energy harvesting device to power other implants or sensors.

However, it is well-known that properties, such as scaffold design, surface topology, chemistry, porosity or the fabrication process are critical to the development of a functioning bone graft, besides good biocompatibility of materials. In particular, the porosity of biomaterials plays an essential role in the context of osteointegration and osteoconduction and supports the migration of cells, capillary ingrowth and the transport of nutrients to cells [14]. Functional bioinspired designs can be produced by utilizing advanced manufacturing techniques, such as electrospinning, freeze casting, sol-gel-techniques or additive manufacturing [15,16]. In particular, additive manufacturing, such as binder jetting, selective laser sintering or extrusion-based techniques became increasingly attractive based on their broad versatility and the ability to fabricate freely designed and patient-specific geometries [17,18]. The application of piezoelectric ceramics as a biomaterial processed via additive manufacturing represents a promising and novel approach in biomaterial manufacturing. Most experiments on biocompatible piezoceramics have so far been carried out using slip casting or freeze casting, which generate porosity rather randomly and uncontrolled and are limited in their design [8,9,19,20]. Therefore, additive manufacturing represents a promising approach to fabricate complex, defect-specific structures with enhanced osteogenic properties. There are benefits in combining the main aspects of implants for bone tissue engineering, such as a bioinspired, bone mimicking and, in future, patient-specific design with good biocompatibility and enhanced bone stimulating properties due to the piezoelectric effect (Figure 1), can be a promising approach for advanced tissue engineered constructs. In this study, we present non-toxic, piezoelectric and highly porous BaTiO_3_/HA scaffolds by using binder jetting. The scaffolds were described carefully by investigating mechanical and piezoelectric properties, as well as porosity, composition and cytotoxicity.

## 2. Materials and Methods

### 2.1. Materials

BaTiO_3_ powder (Sigma-Aldrich/ Merck KGaA, Darmstadt, Germany), as the main piezoelectric component, with an average particle size d_50_ of <3 µm was used to create the 3D printed scaffolds. To support the osseointegration of the scaffold, spray-dried hydroxyapatite powder (HA19, BioCer Entwicklungs-GmbH, Bayreuth, Germany) with an average grain size d_50_ of ~40 µm was added to create the BaTiO_3_/HA powder blend [21,22]. To enable the 3D printing process, Polyethylenmethacrylate (PEMA, DEGACRYL^©^) provided by Evonik (Evonik Industries, Essen, Germany) was added. The polymer phase forms the backbone of the scaffold after 3D printing and binds the ceramic particles.

### 2.2. Fabrication

**Figure 2:**
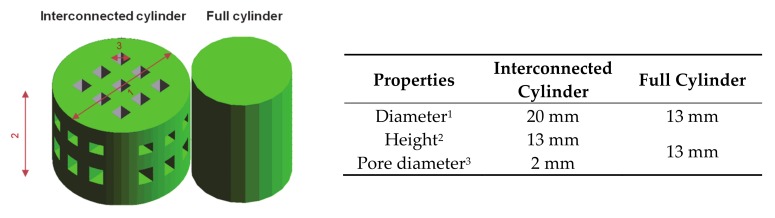
CAD Design and geometrical data of BaTiO_3_/HA composite scaffolds.

In the following study, a composition containing 68 wt.% BaTiO_3_, 18 wt.% HA and 14 wt.% PEMA, featuring high flowability crucial for powder-based printing was used [23]. The resulting material mixture will be called BaTiO_3_/HA composite. The composite powder was homogenized in a laboratory blender for 10 min. For the experiment, different cylindrical samples, dense and with interconnected macropores (Figure 2), were designed with CAD software (SolidWorks 2016 SP 5.0, Dassault systems, Waltham, USA). Afterwards, the composite powder was 3D printed on a commercially available 3D printer Voxeljet VX500 (Voxeljet AG, Friedberg, Germany). The printer deposits a binder fluid (SOLUPOR, Voxeljet AG, Friedberg, Germany) layer-by-layer and partially dissolves the polymeric phase to glue the ceramic particles together. The binder system is a solvent mixture consisting of hexane-1-ol, 2-ethylhexyl acetate and hexyl acetate [24]. After waiting a period of 24 h, the scaffolds were removed from the powder bed. The samples were then stored for at least 24 h in a drying cabinet (Heraeus T6060, Hanau, Germany) at 40 °C.

### 2.3. Thermal Post-Treatment

**Figure 3:**
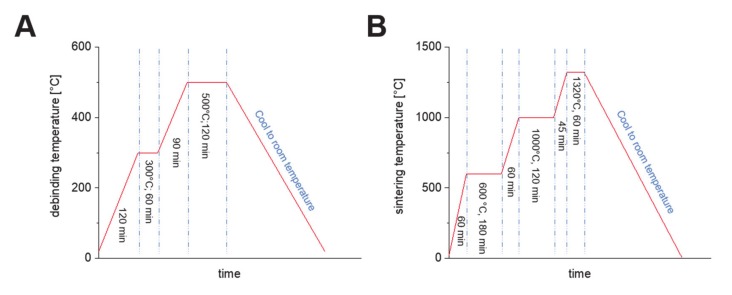
Applicated debinding, (**A**) and sintering curve, (**B**) for 3D printed BaTiO_3_/HA composite scaffolds.

Thermal post-treatment was performed to remove the polymeric phase from the samples and to solidify and densify the composite ceramic. The polymeric phase was removed via pyrolysis from the scaffolds (debinding) by heat-treating in a debinding furnace (L9R, Nabertherm GmbH, Lilienthal, Germany) at 300 °C for 1 h and at 500 °C for 2 h (Figure 3A). In a final step, the debound green parts were subjected to a subsequent sintering treatment at 600 °C, 1000 °C and 1320 °C at atmosphere (Figure 3B).

### 2.4. Polarization and Piezoelectric Characterization

To achieve piezoelectric properties, the fabricated scaffolds were polarized in a strong electrical field. The polarization setup consists of a sample holder with silver (Ag) electrodes on both sides that were connected to a high voltage power supply (HNCs 10000-180 pos., Heinzinger, Rosenheim, Germany) resembling the design of a capacitor. The sample holder was placed in a heated silicon oil bath to prevent any sparks and to polarize the scaffolds shortly beyond the Curie temperature. For the polarization, different settings were applied. To find the best polarization parameters, the field strength, polarization time and polarization temperature were altered in 4 steps starting from 0.667 kV/mm to 1.25 kV/mm. The piezoelectric constant d_33_ of different polarized scaffolds (n = 5 samples for each group, full cylinder) was measured with the Berlincourt method using a d_33_ piezometer (PM300, PIEZOTEST, Singapore).

### 2.5. Shrinkage, Microstructure, Porosity and Mechanical Characterization

The shrinkage of the scaffolds after 3D printing and after sintering was determined by using a digital caliper (conforms to DIN 682) measuring the diameter and height (n = 5). For mechanical characterization, compression tests on full cylindrical specimens (n = 5) were performed using a uniaxial testing machine (Zwick Roell Z5.0, ZwickRoell GmbH, Ulm, Germany) with a 5 kN load cell and a crosshead speed of 0.5 mm·min^−1^. Prior to the compression test, the top and bottom surfaces of the scaffolds were grinded to achieve optimal testing conditions in a uniaxial testing state. The porosity of final 3D printed BaTiO_3_/HA scaffolds (full cylinders) was measured using a microCT scanner (Skyscan 1076, Bruker, Kontich, Belgium) with a source voltage of 96 kV and a source current of 102 µA (n = 3). The samples were scanned with a resolution of 9 µm/ voxel in a 360° scan taking an average of 4 frames every 0.6° scan increment to improve image quality and reduce noise. To further reduce beam hardening an aluminum filter of 0.5 mm was used. For the reconstruction, the Software NRecon (Version: 1.6.10.4, Bruker, Kontich, Belgium) was used. By applying the Feldkamp algorithm with a Gaussian smoothing and a beam hardening reduction (80%) and ring artefact reduction (18 of 20) the cross-sectional images were reconstructed. Segmentation of the microCT data was performed using a global histogram threshold of 65 (lower limit) and 255 (upper Limit) to binarize the scans thoroughly to run a 3D analysis script provided by the software CT Analyzer (Version: 1.15.4.0, Bruker, Kontich, Belgium). Subsequently, morphometric properties of the scaffolds were calculated. To further investigate the microstructure and the elementary composition of the printed and sintered composite scaffolds, a scanning electron microscope (SEM) Merlin VP compact (Carl Zeiss AG, Jena, Germany) coupled with an electron dispersive X-ray spectroscopy (EDX) detector XFlash 6/30 Co. (Bruker, Berlin, Germany) was used. The structural composition was investigated by means of X-ray diffraction (XRD). XRD powder pattern were recorded on a Panalytical X’Pert diffractometer (PANalytical GmbH, Almelo, Netherlands) equipped with an Xcelerator detector using automatic divergence slits and Cu kα_1_/α_2_ radiation (40 kV, 40 mA; λ = 0.15406 nm, 0.154443 nm). Cu beta-radiation was excluded using a nickel filter foil. The measurements were performed in 0.0167° steps. Every single point of the diffraction data was collected for 25 s (starting materials) or 100 s (after thermal treatment). The samples were mounted on silicon zero background holders. The obtained intensities were converted from automatic to fixed divergence slits (0.25°) for further analysis. The peak positions and profile were fitted with Pseudo-Voigt function using the HighScore Plus software package (Version 3.0, PANalytical GmbH, Almelo, Netherlands). Phase identification was undertaken by using the PDF-2 2016 database of the International Center of Diffraction Data (ICDD).

### 2.6. In Vitro Characterization

#### 2.6.1. Cell Culture and Maintenance

Mouse calvaria pre-osteoblast MC3T3-E1 cells (Sigma Aldrich, Taufkirchen, Germany) were cultured according to manufacturer recommendations. In brief, cells were cultured in T-75 flasks (Sarstedt, Nümbrecht, Germany) until maximum passage of p16 using Dulbecco’s Modified Eagle Medium (DMEM, ThermoFisher Scientific, Dreieich, Germany) supplemented with 10% (v/v) fetal calf serum (FCS, ThermoFisher Scientific, Dreieich, Germany) and 1% (v/v) penicillin-streptomycin (PS, Sigma Aldrich, Taufkirchen, Germany) cell culture medium at a humidified atmosphere of 95% air, 5% CO_2_ at 37.5 °C. Cells between passage p5 and p16 were used. For cell experiments, the cells were detached from cell culture flasks using Trypsin/Ethylenediaminetetraacetic acid (Sigma Aldrich, Taufkirchen, Germany) and counted using the Trypan Blue exclusion method.

#### 2.6.2. Indirect In-Vitro Cytotoxicity Test

To assess the materials towards their cytocompatibility, indirect cytotoxicity tests were performed using the eluate-exposure method in line with the ISO 10993-5 standard [25]. Sintered BaTiO_3_/HA scaffolds (n = 6) were sterilized using hot air sterilization at 160 °C for two hours. The scaffolds were immersed for 24 h in cell culture medium (0.2 g/mL) to create the respective scaffold eluates. MC3T3-E1 cells were harvested and seeded in 12-well tissue culture plates (Sarstedt, Nuembrecht, Germany) at a concentration of 100.000 cells.ml-1 for 24 h (100.000 cells/well). Cells were then washed with phosphate-buffered saline (PBS, ThermoFisher Scientific, Dreieich, Germany) and eluates (1 mL) of BaTiO_3_/HA were added to the cells. Cell culture medium and cell culture medium containing 6% (v/v) DMSO served as positive and negative controls, respectively. The cells were incubated for 24 h with scaffold eluates and positive and negative controls before viability analysis.

#### 2.6.3. Direct Cytocompatibility Test

For direct evaluation of the cytocompatibility of the BaTiO_3_ materials, MC3T3-E1 cells were seeded on BaTiO_3_/HA scaffolds (100.000 cells/scaffold, n = 12 scaffolds) in 24-well tissue culture plates (Sarstedt, Nuembrecht, Germany) at a concentration of 100.000 cells.ml-1 and incubated for 24 h in a humidified atmosphere of 95% air, 5% CO_2_ at 37.5 °C, followed by in-vitro cell characterization. Tissue culture polystyrene (TCPS) served as positive controls.

#### 2.6.4. Cell Viability and Proliferation

Water-soluble tetrazolium salt assay (WST-8). Cell viability was assessed using a WST-8 kit (Cell Counting Kit-8, Sigma Aldrich, Taufkirchen, Germany) by conversion of a tetrazolium salt into a water-soluble formazan during cellular metabolism, allowing calorimetric analysis by adsorbing at 450 nm. After 24 h of incubation, cells were washed using PBS and incubated in cell culture medium containing 1% WST-8 solution for three hours. Aliquots of 100 µL of supernatant were pipetted into 96-well plates (Sarstedt, Nuembrecht, Germany) as technical duplicates and the absorbance at 450 nm measured using a multi-well plate reader (Type Phomo, Anthos Mikrosysteme GmbH, Friesoythe, Germany).

Lactate dehydrogenase (LDH). Intracellular and extracellular lactic dehydrogenase (LDH) levels were measured using a lactic dehydrogenase based in vitro toxicology assay kit (TOX7, Sigma Aldrich, Taufkirchen, Germany). Intracellular LDH levels were assessed, in order to analyze the number of cells relative to TCPS positive references. Extracellular LDH was measured as an indicator of cell death in direct cytocompatibility tests. For Intracellular LDH determination, cells were washed with PBS and permeabilized using 1/10% (v/v) of LDH assay lysis solution in H_2_O for 30 min at room temperature (22 °C, RT). Next, 500 µL aliquots of lysate were frozen at −21 °C until further use. Lysates were then centrifuged and 60 µL of lactate dehydrogenase assay mixture (LDH, equal volumes of LDH assay substrate solution, assay dye and cofactor) were added to 140 µL of lysate sample inside polystyrene cuvettes (pathlength 10 mm). The mixture was incubated in the dark for 30 min at RT. The reaction was quenched using 500 µL of 1N HCL. Absorbance values at 490 nm and 690 nm were recorded using a UV/Vis spectrophotometer (Specord 40, Analytik Jena AG, Jena, Germany), subtracting the absorbance of 690 nm from the absorbance at 490 nm for final analysis. For extracellular LDH analysis, aliquots of cell culture medium supernatant (500 µL) were directly withdrawn from the cell culture, centrifuged, and 140 µL of supernatant processed analog to intracellular LDH measurements.

#### 2.6.5. Fluorescence Microscopy

LIVE/DEAD staining. Calcein acetoxymethyl ester Calcein AM and propidium iodide (PI) (both Invitrogen, Carlsbad, CA, USA) stainings were performed on MC3T3-E1 cells indicating live, and dead cells, respectively. In brief, cells were incubated in a master stock solution of Hanks’ Balanced Salt Solution (HBSS) containing 4 µL ml-1 and 1 µL ml-1 Calcein AM (4 µM) and PI (1.5 µM) solution for 45 min. Cells on PS or BaTiO_3_ scaffolds were washed using HBSS and fixed using a fixing solution containing 7.4 g paraformaldehyde (Sigma Aldrich, Taufkirchen, Germany), 6.048 g piperazine-N,N′-bis(2-ethane sulfonic acid) (PIPES) (Merck, Darmstadt, Germany), 0.076 g ethylene glycol tetraacetic acid (EGTA) (Sigma Aldrich, Taufkirchen, Germany) and 8 g polyethylene glycol (PEG) (Sigma Aldrich, Taufkirchen, Germany) in 200 mL HBSS, adapted to a pH of 7.4 using NaOH (VWR, Darmstadt, Germany). Images of the cells present on polystyrene were taken using an inverse fluorescence microscope (Scope.A1, Carl Zeiss, Oberkochen, Germany), while scaffolds were imaged using an upright fluorescence microscope. The area of live cells was quantified using n ≥ 4 biological replicates with a minimum of three images measuring the calcein-AM 488 green fluorescent area on identically sized images (1388 × 1038 pixel) using the Fiji ImageJ (version 1.52i) plugin. In direct cytocompatibility tests, cell nuclei were additionally stained using DAPI (Invitrogen, Carlsbad, CA, USA).

#### 2.6.6. Cell Material Interaction Using Scanning Electron Microscopy (SEM)

The interaction of MC3T3-E1 cells with BaTiO_3_/HA scaffolds was assessed using scanning electron microscopy. Cells were fixed after incubation on the samples and incubated in SEM-fixing solutions for one hour, respectively., The samples were then dehydrated using an ethanol series by incubation inside 30%, 50%, 70%, 75%, 80%, 85%, 90%, 95% and 99% EtOH/H_2_O solutions for 10 min each. Prior to imaging, the samples were dried using a critical point drier (EM CPD300, Leica, Wetzlar, Germany). SEM images were recorded using an Auriga CrossBeam unit (Carl Zeiss, Oberkochen, Germany).

#### 2.6.7. Statistical Analysis

All experiments were carried out with at least four replicate samples per condition. Statistical analysis of in-vitro cell experiments (quantification of live cells, WST-8 viability measurement, LDH level) was carried out using one-way analysis of variances (ANOVA) with posthoc Bonferroni multiple comparison tests using the Origin software (Version: 2019, OriginLab Corporation, Northampton, MA, United States). A pairwise comparison between two groups was performed using Welch’s t-test. All groups were exposed to a shapiro-wilk normality test prior analysis. Data that is not normally distributed (extracellular LDH level, direct biocomp. test) was analyzed using a non-parametric Mann-Whitney U test using GraphPad Prism 8.0 statistical software (GraphPad Software, San Diego, CA, United States). Homogeneity of variances was ensured using Brown-Forsythe criteria for ANOVA analyses. Data is reported as mean ± standard deviation (SD). *,** and *** indicate statistical significant differences with p < 0.05, 0.01 and 0.001, respectively.

## 3. Results and Discussion

### 3.1. Characterization of BaTiO_3_/HA Raw Material

**Figure 4:**
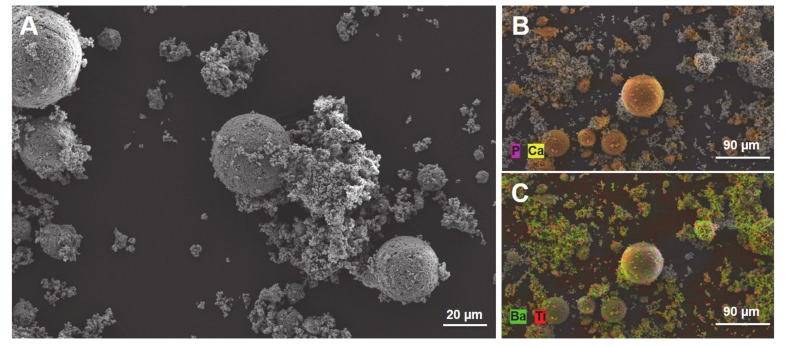
SEM image of the BaTiO_3_/HA powder mixture used for the 3DP process (**A**, scale bar: 20 µm). Elementary classification by EDX spectroscopy for HA (**B**, scale bar: 90 µm) and BaTiO_3_ (**C**, Scale bar: 90 µm).

Figure 4 shows the SEM + EDX images of the BaTiO_3_/HA powder mixture used to print BaTiO_3_/HA scaffolds. The particle size and morphology of HA particles can be strongly distinguished from the particles of BaTiO_3_ (Figure 4A). HA particles show a spherical morphology with a particle size varying between 20–50 µm. In comparison, the BaTiO_3_ raw materials consist of much smaller particles with a d_50_ of 3 µm in a more polygonal shape arranged to agglomerates. The addition of HA to the printing process allowed an increase in the flowability of the powder mixture, which can be associated with significantly higher particle size and spherical particle shape. In this case, HA not only works as a bone-mimicking biomaterial, but it also serves as a flowability agent, thereby, facilitating the 3D printing process.

### 3.2. 3D Printing, Scaffold Morphology and Composition of BaTiO_3_/HA

**Figure 5:**
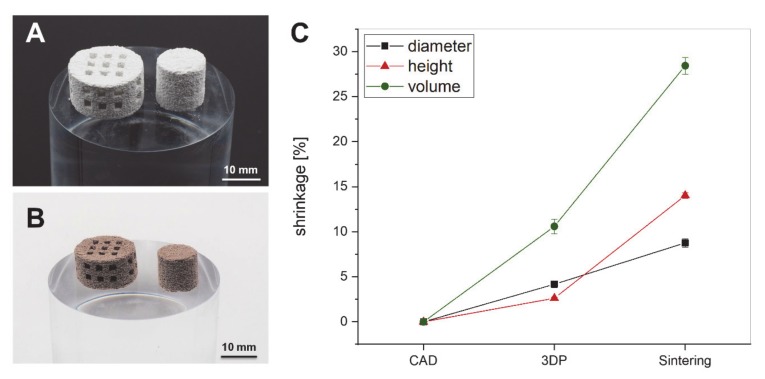
Three-dimensional (3D) printed cylindrical and interconnected scaffolds from BaTiO_3_/HA composite prior to debinding and sintering (**A**, scale bar: 10 mm) and afterward (**B**, scale bar: 10 mm). The shrinkage of the BaTiO_3_/HA scaffolds classified into fabrication process (3DP) and sintering, representing the fidelity of the printing process and the impact of thermal post-treatment (**C**).

The processing of the BaTiO_3_/HA powder mixture was possible and resulted in the fabrication of interconnected, porous scaffolds with an average macropore size of about 1.5 mm before (Figure 5A,B). After sintering at 1320 °C, the scaffolds show a strong shrinkage of about 28.4 ± 0.93% in volume but maintain their geometrical shape (Figure 5B,C). The volumetric shrinkage at this height is well-known for sintered bioceramics and compares to the shrinkage rates reported in the literature [26,27]. In detail, a slight anisotropic behavior between shrinkage in diameter and shrinkage in height occurs (Figure 5C). Moreover, the figure allows the valuation of printing fidelity with the BaTiO_3_/HA composite material system. The printed scaffolds are slightly smaller in height and diameter than the CAD-designs.

**Figure 6:**
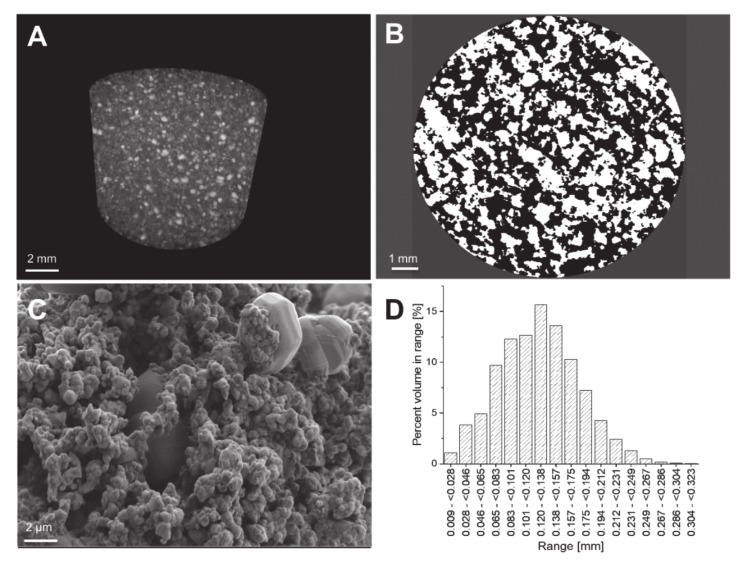
(**A**) Three-dimensional maximum intensity projection (MIP) of a BaTiO_3_/HA scaffolds with visible particles of different densities (scale bar: 2 mm); (**B**) The binarised cross-sectional microCT images reveal the number of pores (black) and provide the basis for a 3D calculation of porosity (scale bar: 1 mm). (**C**) The SEM images underline the results visible in the microCT of a highly porous network of particles which are roughly sintered. Large particles of HA are embedded in a percolating network of BaTiO_3_ particles through the whole scaffold (scale bar: 2 µm); (**D**) The pore size distribution of a 3D printed BaTiO_3_/HA scaffold.

In assessing the microstructure and scaffold morphology of sintered scaffolds by microCT, the distribution of particles and pores throughout the scaffold is revealed (Figure 6A). Larger HA particles are embedded in a matrix of roughly sintered BaTiO_3_ particles (Figure 6C), forming a connected network through the scaffold, to obtain the piezoelectric properties [28]. Such a network is comparable to a percolation matrix, known from nanoparticles [20]. The structural analysis with microCT reveals an open porosity of about 50% of the volume of the scaffold, predominantly resulting from the debinding step when removing the organic matrix. Moreover, the large differences in particle size of HA and BaTiO_3_ and the chosen sintering temperature additionally seem to increase the porosity with a great impact on the physical properties of the scaffolds (Figure 6B) [29]. Nevertheless, the pore size distribution shows a large amount of pores in a range of 100–200 µm which is known to be in a favorable region for osteogenesis (Figure 6D) [14]. In terms of impact on mechanical characteristics, the BaTiO_3_/HA composite scaffold shows a very limited capability to withstand mechanical forces properly. It was not possible to achieve clear data due to the high porosity and the inherent brittleness. The compressive strength of 3D printed BaTiO_3_/HA scaffolds varied in a range of 50–370 kPa, resulting in an average compressive strength of 150 ± 120 kPa. Overall, the scaffolds were easy to manage and survived any transport and treatment. Nevertheless, a future aim for research is increasing the mechanical properties significantly by changing the sintering treatment or composition.

Even if an openly porous network for osteoconduction and osteoinduction would be preferable, it decreases the mechanical and piezoelectrical properties. As shown by the work of Yap et al. (2018), air-filled pores inhibit a homogenous electrical field propagation during the polarization process, and therefore, result in decreased ferroelectric and piezoelectric properties [30].

**Figure 7:**
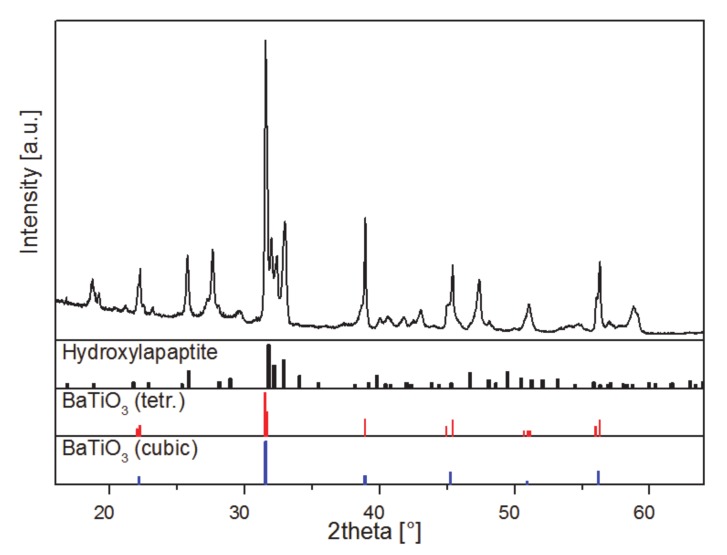
Powder diffraction pattern of 3D printed and sintered BaTiO_3_/HA scaffold and reference data: pdf 01-076-8436 (ICDD, 2016, hydroxyapatite), 01-081-8524 (ICDD, 2016, BaTiO_3_, tetragonal), and 01-081-8527 (ICDD, 2016, BaTiO_3_, cubic), respectively. Reference data is shown in relative intensities. Cu Kα_2_ radiation has been removed arithmetically for clarity.

The phase composition of the 3D-printed and sintered BaTiO_3_/HA-composite is determined by X-Ray diffraction. The pattern, displayed in Figure 7, of the sintered composite, is quite complex, making a distinct identification of the present phases difficult. Due to the elevated sintering temperature of 1320 °C phase transitions, decomposition reactions and ion substitution are very likely to occur [31]. Especially divalent ions, such as the Ca^2+^ ions of the HA are known as a-site modifiers in barium titanate [32]. However, besides HA (pdf 01-076-8436, ICDD, 2016) and the BaTiO_3_ within its cubic modification (pdf 01-081-8524, ICDD, 2016) the tetragonal modification of BaTiO_3_ is identified, which is the most important phase to maintain piezoelectric properties [32]. The XRD patterns of the raw materials are listed in the supplements to demonstrate the purity of the raw materials and the changes to the final composite. Both diffractograms show the high purity of the starting materials for BaTiO_3_ and HA respectively (Figure S1).

### 3.3. Piezoelectric Properties of 3D Printed BaTiO_3_/HA Composites

**Figure 8:**
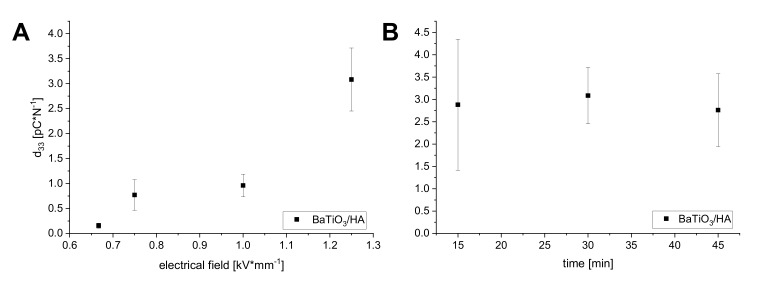
Piezoelectric coefficient d_33_ in dependence of the applied polarization field (**A**) and polarization time (**B**).

To polarize the scaffolds, different polarization conditions were investigated. The domain switching and the achievement of a remanent polarization state in the perovskite phase seems to be clearly linked to the applied electric field strength during the polarization process (Figure 8A). By increasing the field strength up to 1.25 kV/mm, the piezoelectric coefficient d_33_ could be gradually increased up to a maximum of 3.08 pC/N. Applied electrical field strengths of 0.667 kV/mm and below were not able to achieve a sufficient domain orientation resulting in very low d_33_ values of 0.15 pC/N. In order to investigate the impact of polarization time on the piezoelectric properties, the samples were polarized with an electrical field strength of 1.25 kV/mm and a variation of polarization time in three steps. The variation of polarization time revealed no great differences between the different time points (Figure 8B). The results scatter and show a relatively high standard deviation. Nevertheless, a polarization time of 15 min resulted in a comparable average d_33_ of 2.88 ± 1.47 pC/N versus the samples polarized for 30 min with a d_33_ of 3.08 ± 0.63 pC/N and 45 min with a d_33_ of 2.76 ± 0.81 pC/N. A polarization time between 15 and 30 min seems to be sufficient to achieve a stable domain orientation with no further improvement of the piezoelectric constant d_33_ due to longer polarization times. Alteration in the polarization conditions is known to greatly influence the electrical properties of piezoelectric ceramics (electrical field, poling time) [33]. Compared to d_33_ values of pure BaTiO_3_ (150–300 pC/N) reported in the literature [7] the measured d_33_ values of 3D printed BaTiO_3_/HA composites in our study are low, limiting the 3D printed samples for applications such as sensors or energy harvesting. In regard to mimicking the piezoelectric properties of dry bone, which is reported with a d_33_ below 1 pC/N [34,35], the achieved d_33_ values of the 3D printed BaTiO_3_/HA scaffolds could be very satisfying. Very recently, a group around Tang et al. (2017) published comparable data of BaTiO_3_/HA composite mixtures fabricated by slip casting with similar d_33_ values of 1.3 pC/N to 6.8 pC/N (80–90 wt.% BaTiO_3_) and bone-inducing effects compared to pure HA [9]. However, the effect of piezoelectricity on bone remodeling is not fully understood, providing no clear requirements for the beneficial effects of a piezoelectric biomaterial. Therefore, future investigations will aim to clarify how bone cells react to piezoelectric scaffolds in static and dynamic cell cultures.

### 3.4. In Vitro Characterization of BaTiO_3_/HA Composite Scaffolds

#### 3.4.1. Indirect in Vitro Cytotoxicity

**Figure 9:**
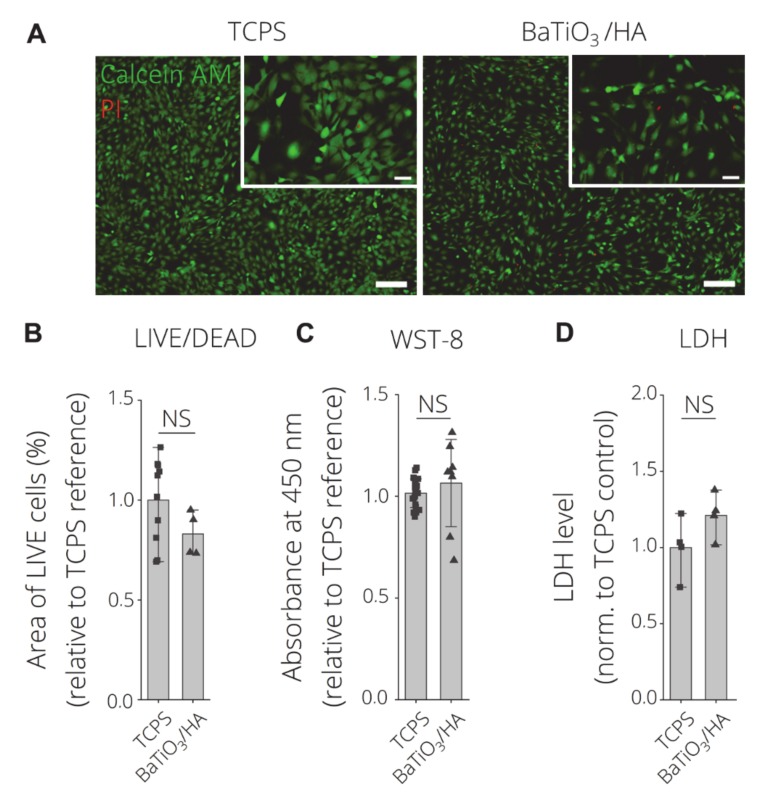
Indirect in-vitro cytotoxicity test according to ISO 10993 using material eluates. (**A**) LIVE/DEAD Images of Calcein AM (green, live) and propidium Iodide (red, dead) stained MC3T3-E1 cells after 24 h of incubation on tissue culture polystyrene (TCPS) with cell culture medium (pos. control), BaTiO_3_/HA scaffolds eluates. Scale bars: 200 µm, 50 µm (detail). (**B**) Quantification of LIVE/DEAD data as area of live cells (%) per FM image (n > 4 biological replicates, n = 3 images) normalized to TCPS reference substrates. (**C**) Indirect cell viability test (WST-8) (n ≥ 4 biological replicates) measured as the absorbance at 450 nm as an indicator for cell viability. (**D**) Intracellular LDH level as a measure of cell death and proliferation (n = 4 biological replicates). Data are shown as mean ±SD. Statistically significant differences were analyzed using one-way ANOVA analysis, with no significant difference indicated (NS).

Figure 9 depicts the viability results of the indirect cell viability test using BaTiO_3_/HA eluates in comparison to tissue culture polystyrene (TCPS) as positive control (DMSO (6%) as neg. ctrl. see Figure S2). Fluorescence microscopy (FM) images of Calcein AM/Propidium iodide LIVE/DEAD staining MC3T3-E1 cells depict majorly live (green) cells with spread morphologies incubated on TCPS and with BaTiO_3_/HA eluates (Figure 9A). Slight less spreading of cells, cultured with BaTiO_3_/HA eluates, is observed. The quantification of the area of live cells per group reveals no statistically significant difference between TCPS and BaTiO_3_/HA samples (Figure 9B), while the sensitivity of MC3T3-E1 cells towards cytotoxic conditions was proven in DMSO 6% conditions (Figure S2) with significantly reduced area of live cells in comparison to both, TCPS and BaTiO_3_/HA. WST-8 viability tests demonstrate no significant difference in viability comparing TCPS and BaTiO_3_/HA samples (Figure 9C). Intracellular LDH levels are in accordance with those findings, showing significantly higher LDH levels in comparison to DMSO 6%, which correlates to the number of cells per condition. Similar to LIVE/DEAD FM and WST-8 results, there is no significant difference in the number of cells comparing TCPS and BaTiO_3_/HA eluate groups. The results indicate no cytotoxic effects of eluates derived from the BaTiO_3_/HA composition that were investigated in this study. Baxter et al. (2009) assessed human osteosarcoma cells on BaTiO_3_/HA substrates similar to those that were 3D printed in our study [36]. The group found no indication of cytotoxicity from their materials [36]. Liu et al. (2016) observed cellular growth on MG-63 cells on BaTiO_3_/HA composite materials of different porosities [37]. Zhang et al. (2014) performed similar eluate tests according to ISO 10993 using L929 cells on BaTiO_3_ based piezo ceramics [8]. Based on 3D printing-processed BaTiO_3_/HA composite, we demonstrate that our tests possesses no in vitro cytotoxic properties, which is in accordance with those previous findings [8,36,37].

#### 3.4.2. Direct in Vitro Cytotoxicity

**Figure 10:**
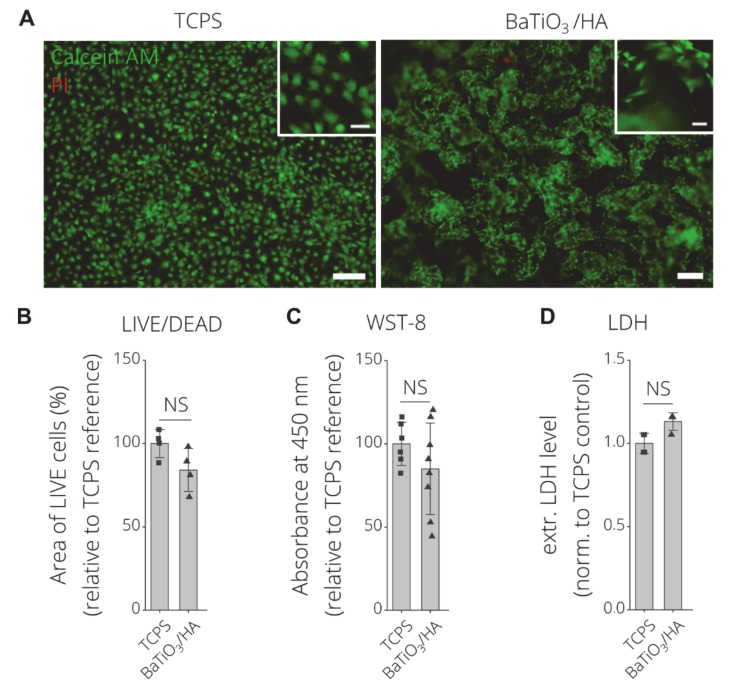
Direct in-vitro cytocompatibility test of MC3T3-E1 cells after 24 h of incubation on BaTiO_3_/HA. (**A**) LIVE/DEAD Images of Calcein AM (green, live) and propidium Iodide (red, dead) stained MC3T3-E1 cells after 24 h of direct incubation on TCPS and BaTiO_3_/HA scaffolds. Scale bars: 200 µm, 50 µm (detail). (**B**) Quantification of LIVE/DEAD data as the area of live cells (%) per image (n > 4 biological replicates, n = 3 images) normalized to tissue culture polystyrene reference substrates. (**C**) Indirect cell viability test (WST-8) (n = 12 biological replicates) measured as the absorbance at 450 nm of metabolized tetrazolim salt to a soluble formazan as an indicator of cell viability. (**D**) Extracellular LDH levels as a measure of cell death, respectively (n ≥ 3 biological replicates). LDH levels with no statistically significant difference were analyzed using the non-parametric Mann-Whitney U test (NS, p < 0.05). Data is shown as mean ±SD. NS indicated no significant difference (p < 0.05) between groups using Welch‘s t-test.

**Figure 11:**
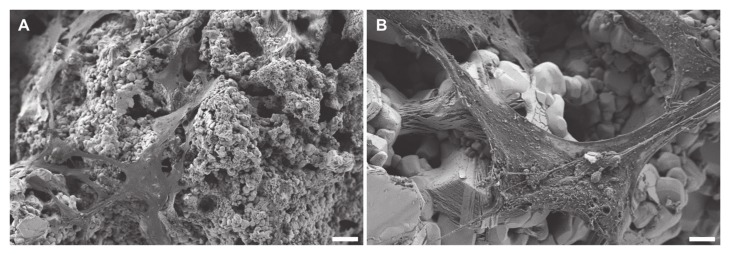
SEM images of MC3T3-E1 cells after 24 h of incubation on BaTiO_3_/HA scaffolds. Representative SEM images of MC3T3-E1 cell-material interaction with BaTiO_3_/HA substrates. Scale bars: 20 µm (**A**), 4 µm (**B**).

Acosta et al. (2017) showed that the interaction of ferroelectric and piezoelectric materials even with a simple cell line (mouse embryonic fibroblasts) is very complex [38]. Figure 10 shows the direct cytocompatibility assessment of MC3T3-E1 seeded for 24 h on 3D printed BaTiO_3_/HA samples. LIVE/DEAD fluorescence images reveal majorly live cells on both, TCPS and BaTiO_3_/HA substrates (green fluorescence) (Figure 10A). MC3T3-E1 cells show attachment and high viability on the 3D printed BaTiO_3_/HA substrate, with no statistically significant difference between TCPS and BaTiO_3_/HA in area of live cells (Figure 10B), viability (WST-8) and extracellular LDH level (Figure 10C,D). Scanning electron microscopy micrographs show spread cell morphologies of MC3T3-E1 cells and attachment on the BaTiO_3_/HA surfaces (Figure 11A), similar to non-printed substrates as described before [36,37]. Liu et al. (2016) observed the spreading of MG-63 cells in porous BaTiO_3_/HA composites, easily spreading over gaps exceeding 10 µm [37]. We observe the same potential of wide spreading morphologies and cell-material interaction of MC3T3-E1 cells on 3D printed BaTiO_3_/HA scaffolds, bridging over surface roughness introduced by the printing process (Figure 11A,B, Figure S3).

Park et al. (1981) showed good interaction of BaTiO_3_ piezoelectric ceramic materials with hard tissues, especially bone and increased osteoblast numbers close to BaTiO_3_ in vivo [13]. Tang et al. (2017) investigated primary osteoblast interaction with BaTiO_3_/HA of different HA loadings recently [9]. We observed cellular adhesion similar to those previous studies, suggesting that the 3D printing process of BaTiO_3_/HA, shown in our study, presents microstructural features and chemical compatibility suitable for osteoblast-like cell attachment and growth, as presented in previous works [9,13,37], proved the cytocompatibility of our 3D printing approach towards BaTiO_3_/HA ceramics.

## 4. Conclusions

Porous, interconnected 3D scaffolds composed of BaTiO_3_/HA were fabricated utilizing a binder jetting process. The fabricated scaffolds possess piezoelectric properties demonstrated by a d_33_ in a comparable range to dry bone. In terms of in vitro evaluation, MC-3T3 pre-osteoblast cells showed a widespread attachment on the surface of the material. LIVE/DEAD screening analysis revealed the high cytocompatibility of the material. Besides the promising results, the study also demonstrates that the fabricated scaffolds exhibit high microporosity through weak mechanical properties. Future investigations will focus on eliminating these disadvantages by a deeper investigation of the microstructure, the sintering behavior and alteration of the material composition. The addition of further bioactive phases to the ceramic powder mixture will be investigated to tailor the bioactivity of the scaffolds and to potentially allow tailoring of the interface of BaTiO_3_/HA/X scaffolds to achieve increased mechanical performance. We show that the additive manufacturing of lead-free piezoelectric BaTiO_3_-based ceramics represents a promising approach to yield scaffolds of designed porosity, equipped with piezoelectric properties for enhanced bone regeneration.

## Supplementary Materials

The following are available online at https://www.mdpi.com/1996-1944/13/7/1773/s1, Figure S1: Powder diffraction patterns of the ceramic raw materials hydroxyapatite (**A**) and BaTiO_3_ (**B**). Both patterns are in good agreement with the ICDD reference data 01-076-8436 (ICDD, 2016, hydroxyapatite), 01-081-8524 (ICDD, 2016, BaTiO_3_, tetragonal) and 01-081-8527 (ICDD, 2016, BaTiO_3_, cubic), respectively. Figure S2: Indirect in-vitro cytotoxicitiy test according to ISO10993 using material eluates. (**A**) LIVE/DEAD Images of Calcein AM (green, live) and propidium Iodide (red, dead) stained MC3T3-E1 cells after 24 h of incubation in DMSO (6%) (neg. control). Scale bars: 200 µm, 50 µm (detail). (**B**) Quantification of LIVE/DEAD data as area of live cells (%) per FM image (n > 4 biological replicates, n = 3 images), Indirect cell viability test (WST-8) (n ≥ 4 biological replicates) measured as the absorbance at 450 nm as an indicator for cell-viability and Intracellular LDH level as a measure of cell death and proliferation (n = 4 biological replicates), all normalized to the tissue culture polystyrene reference (TCPS) control. Data are shown as mean ±SD. *, ** and *** indicate statistical significant differences with p < 0.05, 0.01 and 0.001 respectively in comparison to TCPS control using one-way ANOVA analysis. Figure S3: SEM images from the direct cytocompatibility test in a lower magnification showing widely spread cells over the BaTiO_3_/HA composite (scale bar: 10 µm).
