# Supplementary material for: 3D Printing of Piezoelectric Barium Titanate-Hydroxyapatite Scaffolds with Interconnected Porosity for Bone Tissue Engineering
Source: Materials (Basel). 2020 Apr 9;13(7):1773. doi: 10.3390/ma13071773 (PMC7179021; doi:10.3390/ma13071773)
Supplement: Supplementary file 1 [file materials-13-01773-s001.pdf]

Article

3D Printing of Piezoelectric Barium Titanate-Hydroxyapatite Scaffolds with Interconnected Porosity for Bone Tissue Engineering

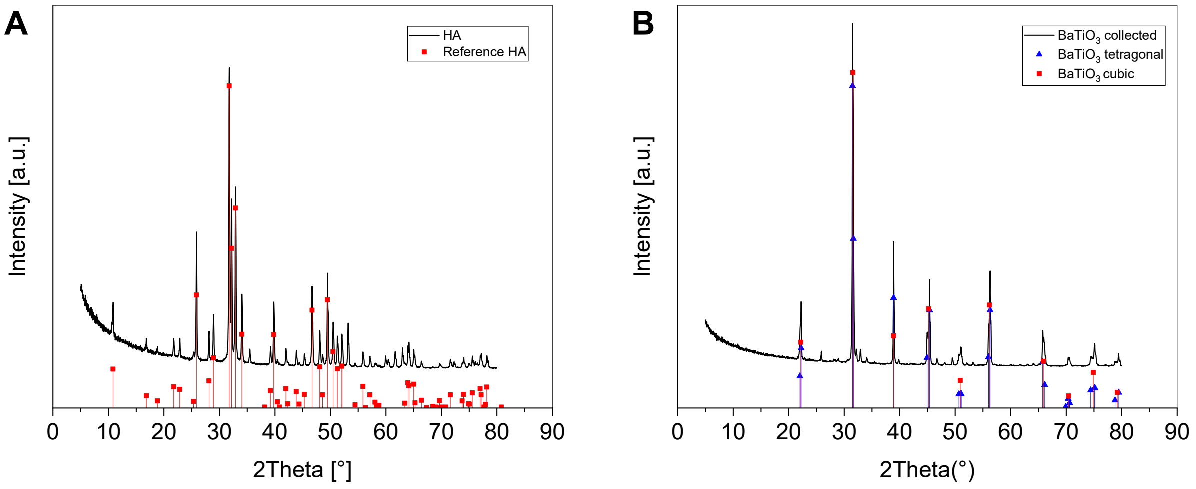

Figure S1. Powder diffraction patterns of the ceramic raw materials hydroxyapatite (**A**) and $BaTiO_3$ (**B**). Both patterns are in good agreement with the ICDD reference data 01-076-8436 (ICDD, 2016, hydroxyapatite), 01-081-8524 (ICDD, 2016, $BaTiO_3$, tetragonal) and 01-081-8527 (ICDD, 2016, $BaTiO_3$, cubic), respectively.

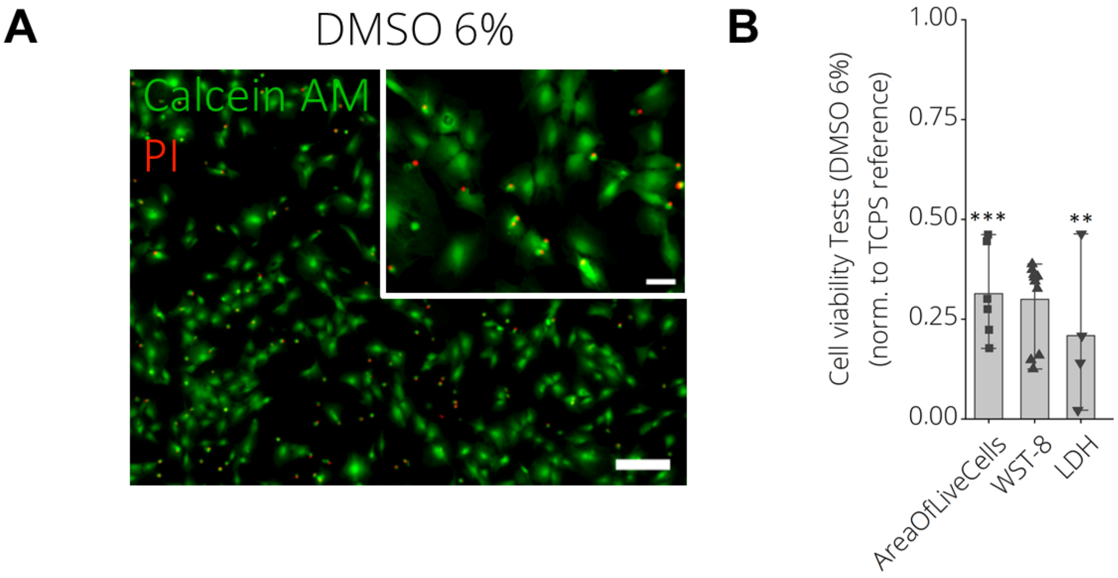

Figure S2. Indirect in-vitro cytotoxicitiy test according to ISO10993 using material eluates. (**A**) LIVE/DEAD Images of Calcein AM (green, live) and propidium Iodide (red, dead) stained MC3T3-

E1 cells after 24 h of incubation in DMSO (6%) (neg. control). Scale bars: 200 μm, 50 μm (detail). (**B**) Quantification of LIVE/DEAD data as area of live cells (%) per FM image (n > 4 biological replicates, n = 3 images), Indirect cell viability test (WST-8) (n ≥ 4 biological replicates) measured as the absorbance at 450 nm as an indicater for cell-viability and Intracellular LDH level as a measure of cell death and proliferation (n = 4 biological replicates), all normalized to the tissue culture polystyrene reference (TCPS) control. Data are shown as mean ±SD. *, ** and *** indicate statistical significant differences with $p < 0.05$, 0.01 and 0.001 respectively in comparison to TCPS control using one-way ANOVA analysis.

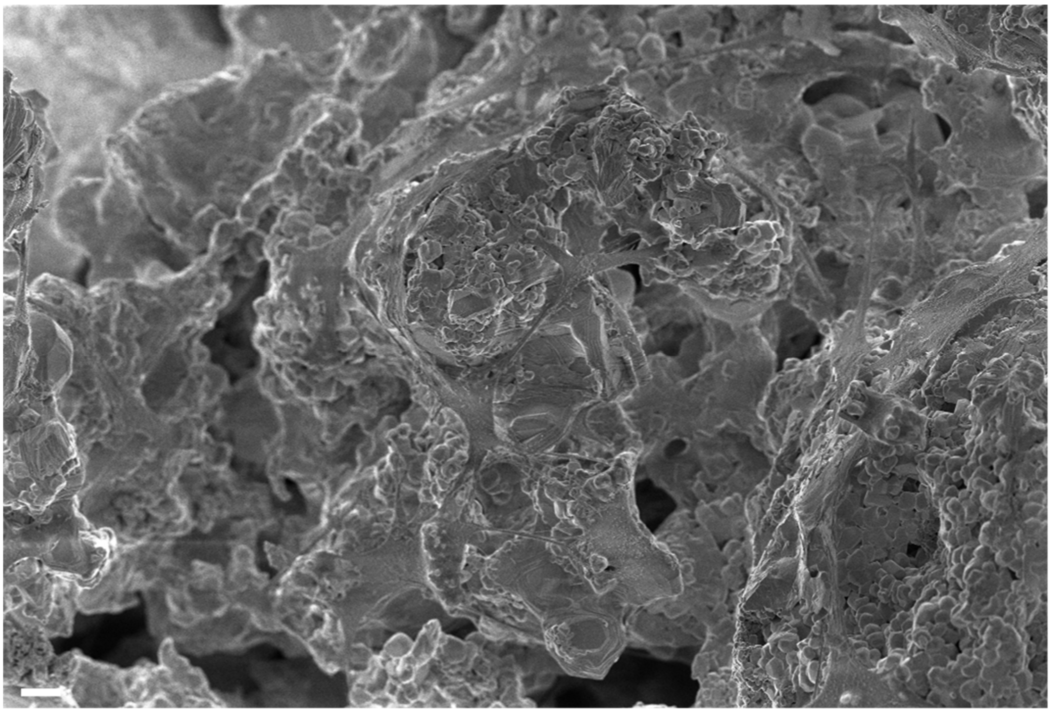

Figure S3. SEM images from the direct cytokompatibility test in a lower magnification showing widely spreaded cells over the $BaTiO_3$/HA composite (scale bar: 10 μm).
